# Use of Random Forest to Predict Adherence in an Online Intervention for Depression Using Baseline and Early Usage Data: Model Development and Validation on Retrospective Routine Care Log Data

**DOI:** 10.2196/53768

**Published:** 2024-11-15

**Authors:** Franziska Wenger, Caroline Allenhof, Simon Schreynemackers, Ulrich Hegerl, Hanna Reich

**Affiliations:** 1 Clinic for Psychiatry, Psychosomatics and Psychotherapy University Hospital Goethe University Frankfurt Frankfurt am Main Germany; 2 Depression Research Centre German Depression Foundation Leipzig Germany; 3 Depression Research Centre German Depression Foundation Frankfurt Germany

**Keywords:** depression, adherence, machine learning, digital interventions, random forest, iFightDepression, iFD, online intervention

## Abstract

**Background:**

Online interventions, such as the iFightDepression (iFD) tool, are increasingly recognized as effective alternatives to traditional face-to-face psychotherapy or pharmacotherapy for treating depression. However, particularly when used outside of study settings, low adherence rates and the resulting diminished benefits of the intervention can limit their effectiveness. Understanding the factors that predict adherence would allow for early, tailored interventions for individuals at risk of nonadherence, thereby enhancing user engagement and optimizing therapeutic outcomes.

**Objective:**

This study aims to develop and evaluate a random forest model that predicts adherence to the iFD tool to identify users at risk of noncompletion. The model was based on characteristics collected during baseline and the first week of the intervention in patients with depression.

**Methods:**

Log data from 4187 adult patients who registered for the iFD tool between October 1, 2016, and May 5, 2022, and provided informed consent were statistically analyzed. The resulting data set was divided into training (2932/4187, 70%) and test (1255/4187, 30%) sets using a randomly stratified split. The training data set was utilized to train a random forest model aimed at predicting each user’s adherence at baseline, based on the hypothesized predictors: age, self-reported gender, expectations of the intervention, current or previous depression treatments, confirmed diagnosis of depression, baseline 9-item Patient Health Questionnaire (PHQ-9) score, accompanying guide profession, and usage behavior within the first week. After training, the random forest model was evaluated on the test data set to assess its predictive performance. The importance of each variable in predicting adherence was analyzed using mean decrease accuracy, mean decrease Gini, and Shapley Additive Explanations values.

**Results:**

Of the 4187 patients evaluated, 1019 (24.34%) were classified as adherent based on our predefined definition. An initial random forest model that relied solely on sociodemographic and clinical predictors collected at baseline did not yield a statistically significant adherence prediction. However, after incorporating each patient’s usage behavior during the first week, we achieved a significant prediction of adherence (*P*<.001). Within this prediction, the model achieved an accuracy of 0.82 (95% CI 0.79-0.84), an F1-score of 0.53, an area under the curve of 0.83, and a specificity of 0.94 for predicting nonadherent users. The key predictors of adherence included logs, word count on the first workshop’s worksheet, and time spent on the tool, all measured during the first week.

**Conclusions:**

Our results highlight that early engagement, particularly usage behavior during the first week of the online intervention, is a far greater predictor of adherence than any sociodemographic or clinical factors. Therefore, analyzing usage behavior within the first week and identifying nonadherers through the algorithm could be beneficial for tailoring interventions aimed at improving user adherence. This could include follow-up calls or face-to-face discussions, optimizing resource utilization in the process.

## Introduction

Depression is one of the most common mental illnesses worldwide, currently affecting approximately 280 million people, according to the Global Burden of Disease tool by the Institute for Health Metrics and Evaluation [1]. By 2030, it is projected to be one of the leading causes of the burden of disease in high-income countries, such as Germany, where it is expected to rank first in disability-adjusted life years [2]. Despite the significant impairments caused by the disease, a gap persists in the provision of adequate care for depression both globally [3] and within Germany [4]. Multiple factors contribute to the lack of effective care, including limited resources and a shortage of trained health care providers [5]. In Germany, one specific reason for the gap in care is the shortage of available psychotherapy appointments. In 2018, the Federal Joint Committee (Gemeinsamer Bundesausschuss) reported that an additional 2400 health insurance–funded psychotherapy seats were needed to meet demand and reduce wait times for treatment [6]. However, only 776 additional seats were created in the following year [7]. In addition, many patients affected by depression do not primarily turn to a psychotherapist or psychiatrist, but to their respective general practitioner (GP), who may not have the time or professional resources to provide in-depth psychoeducation and subsequent treatment of the symptoms. Thus, more than half of those treated by their GP do not receive treatment with antidepressants or psychotherapy as recommended in the guideline [8]. Digital interventions, such as the iFightDepression (iFD) tool discussed in this paper, are increasingly available as options to help close the gap in effective depression treatment. These tools, based on cognitive behavioral therapy (CBT), enable treatment for a larger number of patients regardless of space and time constraints. A previous meta-analysis indicated that supported internet-based CBTs (iCBTs) can achieve substantial effects, comparable to those of traditional treatments such as antidepressant pharmacotherapy or face-to-face psychotherapy [9].

However, online interventions also come with unique limitations. A major challenge is adherence, defined as the extent to which an individual’s behavior aligns with the recommended treatment plan [10]. For online interventions, adherence also refers to the degree to which users engage with the provided content, as intended by the intervention designers, to achieve optimal progress throughout the program [11]. For example, a meta-analysis on guided iCBT under routine care conditions reported that 73.0% of included patients started the intervention. Of those who began, an average of only 61.3% completed the program as intended [12].

Adherence is crucial for the success of therapy and symptom reduction. Studies on face-to-face treatments have shown that early withdrawal from treatment can lead to prolonged depressive episodes and a higher likelihood of relapse [13,14]. Consequently, how to maintain adherence to interventions such as the iFD tool has become the subject of several studies. These studies have identified sociodemographic and clinical characteristics—such as age, gender, and severity of depressive symptoms—as factors influencing intervention outcomes [15,16]. While younger age and male gender are linked to nonadherence, higher depression severity, older age, and female gender are associated with better adherence, often resulting in greater reductions in depressive symptoms [15,16]. Another factor influencing intervention outcomes is participants’ level of engagement, such as time spent on the tool or the number of modules completed (usage behavior). de Graaf et al [17] found that adherence correlated with depression outcomes at both 3 and 9 months after the intervention. At the 3-month mark, symptom improvement was achieved after more than 12 log-ins and over 173 minutes spent online. Previous studies have shown that sociodemographic and usage-related variables can generally serve as predictors of adherence. However, using these variables to predict adherence at the start of an online intervention—enabling early identification of participants who may drop out prematurely and thus have a reduced chance of benefiting from the intervention—has not been extensively explored.

Therefore, this study aims to address the following research questions:

- Can adherence to the iFD tool be predicted using a random forest model with predictors available at the beginning of the intervention, based on information provided through the entry questionnaire?
- How influential is usage behavior within the first week in predicting adherence to the iFD tool?
- Which explanatory variables hold the highest importance for predicting adherence?

## Methods

### Study Design

This study examined adherence to the iFD tool using data collected between October 1, 2016, and May 5, 2022. During this period, the iFD tool was integrated into routine care for treating depression in Germany. All data were collected routinely as part of an ongoing process evaluation and were not specifically gathered for this study (convenience sample). The study was preregistered on AsPredicted [18] on June 14, 2022, under the title “Machine-learning based prediction of adherence in the iFightDepression tool.” Additionally, this manuscript was prepared in accordance with the Transparent Reporting of a Multivariable Prediction Model for Individual Prognosis or Diagnosis + artificial intelligence (TRIPOD + AI) reporting guidelines [19].

### Participants

Participants were users of the iFD tool in Germany who registered between October 1, 2016, and May 5, 2022, after being invited to join the program by their physician or psychotherapist. To be eligible for participation in this study, users had to be 18 years or older and provide informed consent to participate in the ongoing evaluation.

### Ethics Considerations

The evaluation of data was ethically approved by the Ethics Committee at the Faculty of Medicine at the University of Leipzig on May 27, 2016, with the file reference 172-16/ek-14032016. Participants provided informed consent for this evaluation during their registration in the iFD tool and before the start of the first workshop. Participants consented to the analysis, recording, and processing of their data in a deidentified form, with the possibility of publication in scientific studies. They were informed that they could withdraw their consent at any time, without providing a reason and without negative consequences for their ongoing treatment, at which point their data would be discarded. Participants received no compensation for their participation in the iFD tool or its evaluation.

### Intervention

The iFD tool is a guided web-based self-management tool based on CBT for mild to moderate depression. Available in over 15 languages, including German, English, and Arabic, it is provided by the European Alliance Against Depression (EAAD). The tool has been shown to significantly reduce symptoms of depression and improve quality of life compared with an active control condition (web-based progressive muscle relaxation) [20]. It includes 6 core workshops covering various depression-related topics, along with 2 additional workshops focusing on a healthy lifestyle and depression in the workplace. Each workshop consists of written information, worksheets, exercises, and a mood assessment [20]. Worksheets can be completed online or in print form. Throughout the intervention, patients receive guidance from an iFD guide (such as a GP, psychiatrist, psychotherapist, or other health care provider) who invites them to participate in the iFD tool. This paper focuses solely on the 6 core workshops and is limited to German data, as they contain the largest available sample within the iFD tool and are commonly used in routine care in Germany. Further information about its content and development can be found in more detail elsewhere [21,22].

### Measures

Sociodemographic and clinical information used as predictors for adherence were collected via the entry questionnaire at the start of the iFD tool. Sociodemographic information included age (years), self-reported gender (male, female, or diverse [the third option “diverse” was added to the iFD tool in 2021, offering an alternative for those who do not identify to be male or female]), and expectations for the intervention. Clinical variables comprised details about past depression treatments (eg, “What treatment did you receive during a previous depressive episode? [multiple answers possible]: none, pharmacotherapy, psychotherapy, or other”), current depression treatments (eg, “What treatment are you currently receiving? [multiple answers possible]: none, pharmacotherapy, psychotherapy, or other”), confirmed diagnosis of depression (eg, “Have you been diagnosed with depression [by a GP, specialist, or psychologist]?—yes or no”), the profession of the accompanying guide (GP, psychiatrist, psychotherapist, or other health care provider, such as a psychiatric outpatient clinic), and the 9-item Patient Health Questionnaire (PHQ-9) sum score (as detailed below) at the beginning of the intervention.

Expectations regarding the success of the intervention were measured by a sum score based on the results of the entry questionnaire completed by patients before the start of the intervention. This questionnaire included 11 questions concerning the patients’ expectations for the upcoming online intervention, aimed at assessing their motivation. These questions were extracted from the subscale “Allgemeine Behandlungserwartungen” (General Treatment Expectations) listed in the “Fragebogen zur Messung der Psychotherapiemotivation” (Questionnaire for the Measurement of Psychotherapy Motivation) [23]. Each question could be answered using the following options: completely agree, agree, undecided, disagree, and completely disagree. The internal consistency of the variables contributing to the expectation sum score in our sample yielded a Cronbach α of 0.61 (95% CI 0.59-0.63).

The PHQ-9 score was used to assess baseline depression symptom severity. The Patient Health Questionnaire-9 (PHQ-9) is a diagnostic tool that measures depression severity through self-report, incorporating the Diagnostic and Statistical Manual of Mental Disorders, fourth edition (DSM-IV) diagnostic criteria for depression [24]. It has been proven to be a reliable and valid measure of depression severity [24].

In the second step of our analysis, we included patients’ usage behavior within the first week of the intervention as an additional set of predictors. It contained the time spent on the tool (seconds), the number of logs/clicks on the tool, and the word count in the first worksheet, all recorded within the first week. A variance inflation factor analysis revealed no considerable collinearity between these thematically similar variables (time in the first week: 3.73; logs in the first week: 3.59; word count: 1.26) [25].

### Adherence

To be considered adherent, participants had to demonstrate personal progress and active engagement in the intervention, following the recommendations for adherence reporting by Beintner et al [26] to establish common standards. The criterion for personal progress within the iFD tool was met by all users who had worked on at least 2 modules from the 6 available core workshops and actively utilized at least 70% of the material provided in those workshops. This criterion is grounded in the minimum number of workshops necessary to detect progress in the treatment of participants. It is based on the assumption that sudden gains—defined as rapid, large, and stable improvements in symptoms during the intervention [27]—typically occur within the first third of the intervention [28]. When these sudden gains happen early in the intervention, patients tend to experience more significant changes in depressive symptoms and are more likely to respond to the given treatment [28].

The criterion for active engagement was met if a user logged in at least 6 times within 56 days from their first log-in, with each session lasting a minimum of 60 seconds. This 56-day period aligns with the iFD tool’s recommendation to use and interact with at least 1 module per week. Following this recommendation, users would have a total processing time of 42 days to complete all 6 workshops, with the additional 14 days providing extra personal leeway.

A series of chi-square tests, *t* tests, and Wilcoxon-Mann-Whitney *U* analyses were conducted to compare differences between the adherent and nonadherent groups regarding usage and baseline characteristics, as presented in Tables 1 and 2 (see the data under the column “Test Statistics”). A type I error rate of 0.5% was used for these analyses.

### Statistical Analysis

**Figure 1:**
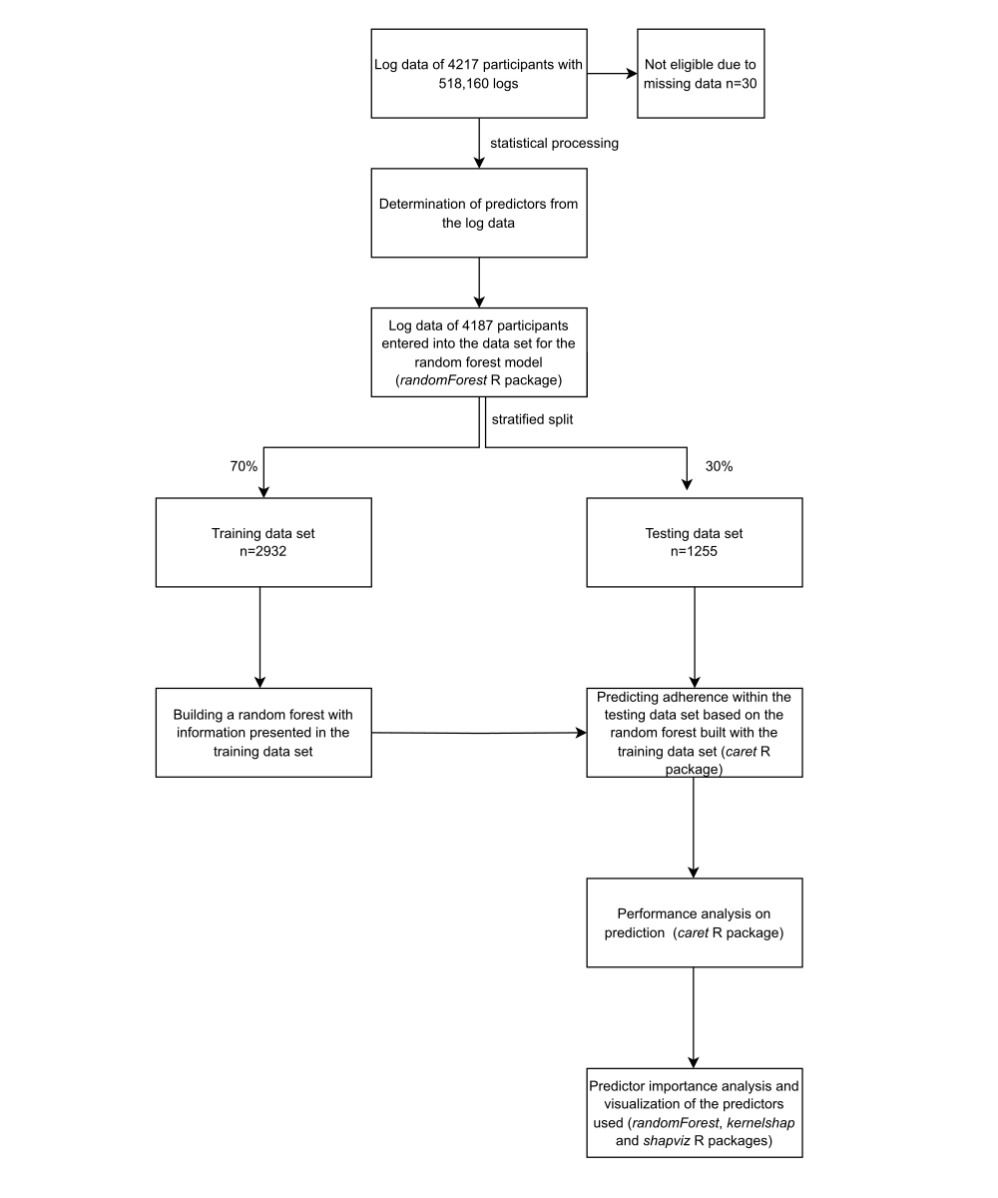
Flowchart showing the data processing procedure for log data of the iFightDepression tool users for model 1 (including sociodemographic and clinical variables) and model 2 (including sociodemographic, clinical, and usage-related variables).

Data were analyzed using R version 4.2.0 (R Foundation). Initially, the data set comprised 518,160 logs from 4217 participants, organized by user ID. It included variables such as age (years), self-reported gender (male, female, or diverse), page ID (an identifier for different pages accessed by the participant during the sessions), event (page with the corresponding page ID visited, responses to questionnaires), and the time stamp of each log/click. From this data set, the outcome measure of adherence (adherent/nonadherent) was derived, and the data were subsequently prepared for analysis. The final data set included predictors for each of the 4187 eligible participants, as 30 participants were excluded from the analysis due to missing data (0.71%). Using this finalized data set, we conducted 2 separate analyses. The first analysis (hereafter referred to as model 1) focused solely on the sociodemographic and clinical predictors. By contrast, the second analysis (referred to as model 2) incorporated all predictors, including usage behavior during the first week. Next, we performed a randomly stratified split of our data set for each analysis, creating a training data set containing 70% (2932/4187) of the participants and a test data set with the remaining 30% (1255/4187) [29]. For the predictions, we constructed a random forest model using the randomForest R package [30], specifying 100 trees (number of trees) and 3 variables to be tried at each split (mtry). We then applied this random forest model to predict participants’ adherence using the caret R package [31] on both the training and test data sets. The same package facilitated the subsequent performance analysis. For predictor importance analyses, we utilized the randomForest R package [30] to create a variable importance plot, the kernelshap R package [32,33] for a subsequent Shapley Additive Explanations (SHAP) analysis, and the shapviz R package [34] for visualizing the SHAP results. The procedure for data processing is illustrated in Figure 1.

The performance of our prediction was assessed using several metrics, including the area under the curve of the receiver operating characteristic (AUC-ROC), AUC of the precision-recall (AUC-PR) curve, sensitivity, specificity, *F*_1_-score, and the out-of-bag (OOB) error rate. The OOB error rate is a method for evaluating the performance of algorithms that utilize bootstrapping, like our random forest, on unseen data. It is calculated using samples that were not part of the bootstrap sample and, therefore, not included in the model’s training process [35]. It allows for unbiased performance estimation and comparison with other random forest algorithms, thereby eliminating the need for additional cross-validation of the data. Furthermore, it enables the evaluation of the algorithm’s performance using different values for our hyperparameters (mtry, node size, and number of trees), facilitating the determination of the optimal combinations of these hyperparameters.

The *F*_1_-score is defined as the harmonic mean between recall (sensitivity) and precision (positive predictive value) [36]. It is commonly used to evaluate data sets with a class imbalance, such as the one in our study. The *F*_1_-score ranges from 0 (worst) to 1 (best) and reflects the model’s ability to identify positive cases (recall) while maintaining accuracy among those cases (precision). While an *F*_1_-score of 1 represents a model that perfectly classifies each observation, an *F*_1_-score of 0 indicates a model that is unable to classify any given observation. Therefore, generally speaking, a higher value of the *F*_1_-score is equivalent to a better model.

The importance of the predictors was assessed using the mean decrease accuracy (the loss of accuracy if this variable is removed from the prediction) and the mean decrease Gini (the average of a variable’s total decrease in node impurity), as well as through a Kernel SHAP analysis. SHAP quantifies the contribution of each feature to the prediction by calculating the average marginal contribution of that feature across all possible subsets of features [34]. It allows for both global interpretations (across all predictions) and local interpretations (for a specific prediction) of the random forest model, and it is grounded in a strong theoretical basis in cooperative game theory [34,37].

For the final validation measures, we conducted 2 sensitivity tests to rule out potential spurious results in our predictions due to biased inputs. First, we examined whether the predictions changed when excluding all participants who met our adherence criteria within the first week of the intervention. Second, we assessed whether the predictions were affected by the low internal consistency of the expectation sum score predictor by excluding this variable. Additionally, we performed several subgroup analyses (see below).

### Random Forest (Machine Learning)

Machine learning, as a branch of artificial intelligence, allows for an objective and automated procedure, thereby minimizing potential subjective influences in the scoring process [38]. To predict participant adherence within the iFD tool, we selected a random forest as the most suitable machine learning model for our approach. The primary reasons for this choice are its applicability and inherent interpretability, which allow for visualization and straightforward implementation. This aligns with our goal of predicting and interpreting adherence in a simple manner based on a limited number of variables available at the start of the intervention. Additionally, the random forest model can achieve accurate predictions with relatively few parameters, effectively handle noisy data (meaningless information that the algorithm cannot interpret), and is less prone to overfitting. Furthermore, compared with the classical approach of using logistic regression, random forest models have demonstrated significantly higher specificity, positive predictive value, *F*_1_-score, accuracy, and prevalence of detection than linear regression in data analyzed in a previous study [39]. In another study addressing a similar question, the random forest model achieved better accuracy than linear regression in 69.0% of all tested data sets (243 in total) [40]. Another advantage of random forest models is their ability to detect nonlinear relationships within given data sets [41].

## Results

### Participants

Table 1 presents the demographic and clinical characteristics of the participants, categorized into 3 groups: all participants, adherent participants, and nonadherent participants. Of the 4187 eligible participants, 1019 were classified as adherent according to our definition, representing 24.34% of registered users. The overall study population was predominantly female (2537/4187, 60.59%), with a mean age of 37.1 years. At the beginning of the intervention, the mean baseline depression severity was moderate, indicated by a PHQ-9 sum score of 13.0. Most participants reported having a confirmed diagnosis of depression (3514/4187, 83.93%). However, only about one-third received pharmacotherapeutic (1179/4187, 28.16%) or psychotherapeutic (1466/4187, 35.01%) interventions for their depression. The majority of participants were referred to the intervention by their psychotherapist (1700/4187, 40.60%), followed by their GP (1311/4187, 31.31%).

It was notable that adherent participants were older than their nonadherent counterparts, had a lower PHQ-9 score at baseline, and expressed higher expectations for the intervention in the entry questionnaire. Additionally, adherers were more likely to have sought treatment for their depression, both currently (at the time of the questionnaire) and in the past.

**Table 1:** Patient summary characteristics assessed via self-report during registration for the iFightDepression tool, categorized by groups (all participants, adherers, and nonadherers).

Variables		All (n=4187)	Adherers (n=1019)	Nonadherers (n=3168)	Test statistics	Effect size
Age in years, mean (SD)^a^		37.1 (13.4)	38.9 (13.7)	36.6 (13.2)	*W*^b^=1452980, *P*<.001	Cohen *d*=0.18 (95% CI 0.11-0.25)
Self-reported gender, n (%)^c^					*χ*^2^ (*df*)=1.296 (2), *P*=.52	η^2^=0.0003
	Female	2537 (60.59)	624 (61.24)	1913 (60.39)	N/A	N/A^d^
	Male	1640 (39.17)	394 (38.67)	1246 (39.33)	N/A	N/A
	Diverse	10 (0.24)	1 (0.10)	9 (0.28)	N/A	N/A
Baseline PHQ-9^e^ score, mean (SD)^c^		13.0 (5.8)	11.3 (5.6)	13.5 (5.7)	*t* test (*df*)=10.81 (1752.5), *P*<.001	Cohen *d*=0.39 (95% CI 0.31-0.46)
Expectations, sum score (SD)^c^		22.5 (3.8)	23.1 (3.6)	22.3 (3.8)	*t* test (*df*)=–6.37 (1788.9), *P*<.001	Cohen *d* 0.22 (95% CI 0.15-0.29)
Diagnosis of depression, n (%)^f^					N/A	N/A
	Present	3514 (83.93)	882 (86.56)	2632 (83.08)	*χ*^2^ (*df*)=6.645 (1), *P*=.009	Φ=0.04
Preceding depression treatments, n (%)^f^					N/A	N/A
	Pharmacotherapy	1179 (28.16)	287 (28.16)	892 (28.16)	*χ*^2^ (*df*)<.001 (1), *P*>.99	Φ=0
	Psychotherapy	1466 (35.01)	365 (35.82)	1101 (34.75)	*χ*^2^ (*df*)=0.339 (1), *P=*.56	Φ=0.01
Current depression treatments, n (%)^f^					N/A	N/A
	Pharmacotherapy	1970 (47.05)	489 (47.99)	1481 (46.75)	*χ*^2^ (*df*)=0.427 (1), *P=*.51	Φ=0.01
	Psychotherapy	2279 (54.43)	588 (57.70)	1691 (53.38)	*χ*^2^ (*df*)=5.644 (1), *P=*.02	Φ=0.04
Guide profession, n (%)^c^					*χ*^2^ (*df*)=13 (1), *P=*.02	η^2^=0.006
	General practitioner	1311 (31.31)	281 (27.58)	1030 (32.51)	N/A	N/A
	Psychiatrist	520 (12.42)	116 (11.38)	404 (12.75)	N/A	N/A
	Psychotherapist	1700 (40.60)	438 (42.98)	1262 (39.84)	N/A	N/A
	Others	488 (11.66)	125 (12.27)	363 (11.46)	N/A	N/A
	Unknown^g^	168 (4.01)	59 (5.79)	109 (3.44)	N/A	N/A

^a^Variable modality: numeric.

^b^*W*: test statistic for the Wilcoxon-Mann-Whitney *U* test.

^c^Variable modality: factor.

^d^N/A: not applicable.

^e^PHQ-9: 9-item Patient Health Questionnaire.

^f^Variable modality: binary.

^g^Unknown: no information available.

### Usage Behavior

**Figure 2:**
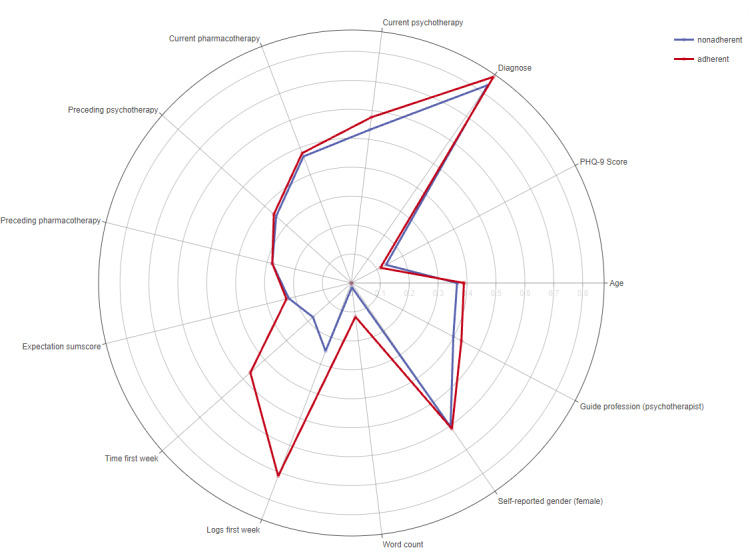
Radar chart showing the main differences in patient characteristics within the iFightDepression tool between the 2 subgroups, adherers and nonadherers. The figure shows proportions for categorial variables and normalized means (decimal scaling) for quantitative variables to allow comparison. PHQ-9: 9-item Patient Health Questionnaire.

Table 2 presents the usage behavior of participants during the first week of the intervention. Among the 3 groups, adherers exhibited higher values across all usage behavior parameters. Figure 2 illustrates the differences between the adherent and nonadherent groups in a radar chart, which represents all variable groups, including sociodemographic, clinical, and usage behavior–related variables.

**Table 2:** Patient summary characteristics of all included iFightDepression tool users regarding usage behavior within the first week of the intervention, sorted by groups (all, adherers, and nonadherers).

Variables			All (n=4187)		Adherers (n=1019)		Nonadherers (n=3168)		Test statistics, *W*^a^ (*P* value)		Effect size, Cohen *d* (95% CI)
Usage behavior within the first week, mean (SD)											
	Word count, first worksheet^b^	39.8 (154.2)		117.7 (269.6)		14.8 (74.1)		822,677 (<.001)		0.7 (0.62-0.77)	
	Logs^b^	36.4 (40.7)		71.4 (58.7)		25.2 (23.8)		649,031 (<.001)		1.3 (1.22-1.38)	
	Time online (minutes)^b^	41.3 (43.9)		77.8 (61.6)		29.6 (27.7)		663,495 (<.001)		1.24 (1.17-1.32)	

^a^*W*: test statistic for the Wilcoxon-Mann-Whitney *U* test.

^b^Variable modality: numeric.

### Prediction Performance and Validation

**Figure 3:**
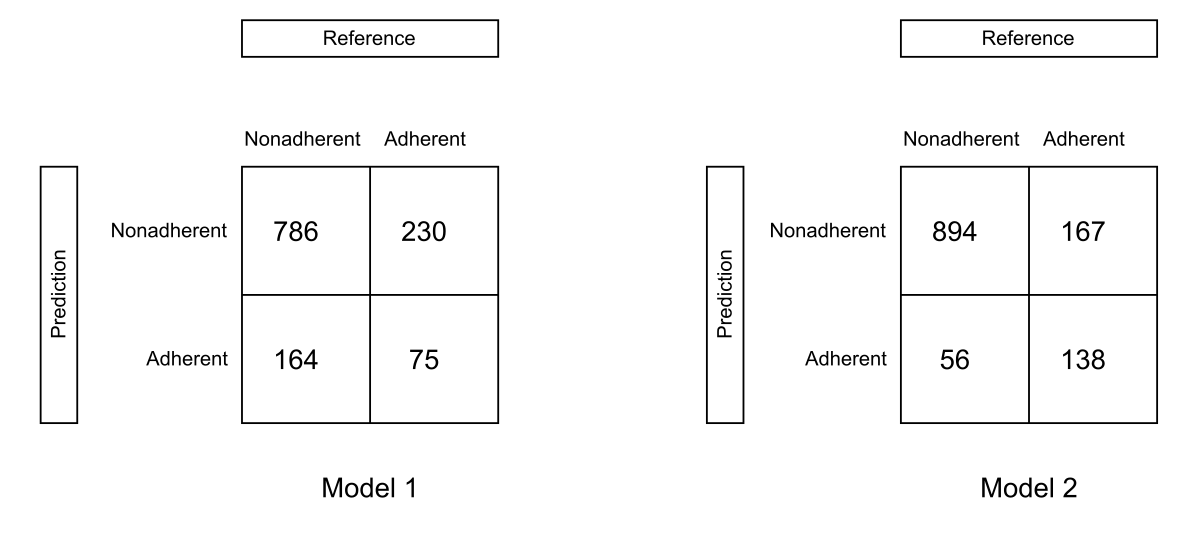
Confusion matrix for both random forest predictions (models 1 and 2) in the test data set (30% of data) depicting the predicted outcome (prediction) versus actual adherence status (reference). Model 1: prediction of adherence based on sociodemographic and clinical variables. Model 2: prediction of adherence adding the usage behavior within the first week.

Figure 3 shows the confusion matrix for both predictions using the test data set (1255/4187, 30% of the data). Model 1 corresponds to the prediction based solely on sociodemographic and clinical predictors, while model 2 also includes usage behavior within the first week. Model 1 misclassified 31.39% (394/1255) of participants in the test data set, resulting in 164 misclassifications in the false-positive classification, meaning 164 out of 1255 patients were predicted to be adherent while actually being nonadherent. In model 2, the proportion of incorrect predictions was lower, with a misclassification rate of 17.77% (223/1255) in the testing data set and a false-positive classification of 56. This reduced the proportion of false-positive predictions from 13.07% (164/1255) to 4.46% (56/1255).

Table 3 demonstrates the performance measures of random forest models 1 and 2, including accuracy, *F*_1_-score, AUC, sensitivity, specificity, positive predictive value, negative predictive value, no information rate, and OOB error rate. The ROC curve is shown in Figure 3.

**Figure 4:**
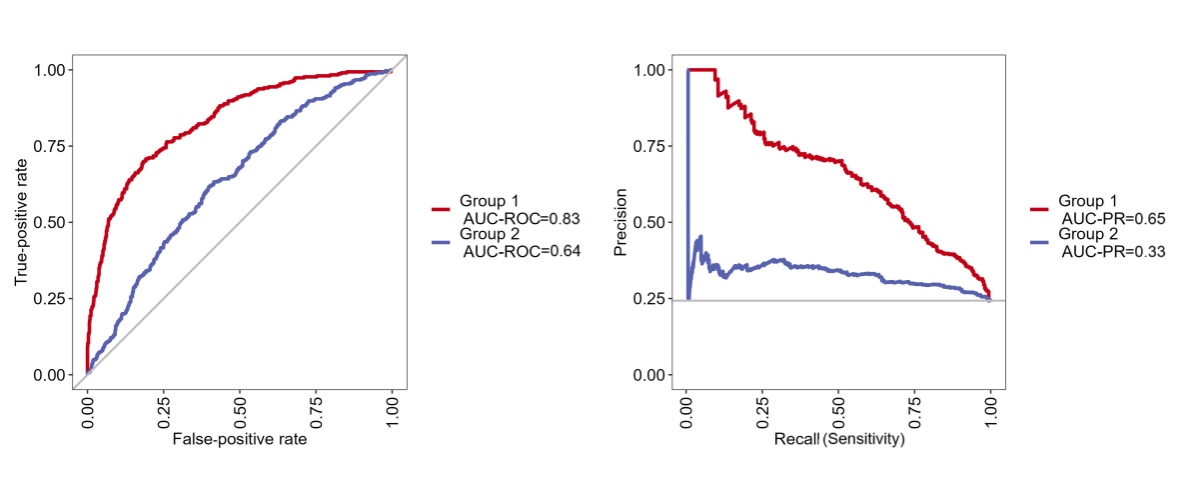
Receiver operating characteristic (ROC) curve and precision-recall (PR) curve for models 1 and 2. Model 1: prediction of adherence based on sociodemographic and clinical variables (blue/group 2). Model 2: prediction of adherence adding the usage behavior within the first week (red/ group 1). AUC: area under the curve.

Within model 1, the algorithm achieved a prediction accuracy of 0.69 (95% CI 0.67-0.71), which was not superior to the no information rate (*P*>.99). In addition to the accuracy, we reported an *F*_1_-score of 0.28 due to the inherent class imbalance. The specificity (true-negative rate) was 0.83, while the sensitivity (true-positive rate) for the first prediction was 0.25. Figure 4 (left side, blue graph) shows the ROC. The AUC calculated from the ROC was 0.64. An AUC score of 0.5 indicates that the model performs no better than random chance. As a result of the class imbalance in our data, we also generated a PR curve, as shown in Figure 4 (right side, blue graph). In this graph, the AUC reached 0.33.

After obtaining nonsignificant results (see above) with the first model, the usage behavior within the first week of the intervention was added as a predictor for the second model. With this additional set of predictors, the algorithm achieved an accuracy of 0.82 (95% CI 0.80-0.84) on the unseen testing data, which proved to be significant with a *P* value of <.001 compared with the no information rate, and an *F*_1_-score of 0.55. The specificity of the prediction was 0.94, and the sensitivity improved to 0.45 compared with model 1. The AUC increased to 0.83 using the ROC and to 0.65 using the PR curve. With an AUC of around 0.70, the model is considered to have good overall performance.

To further validate the algorithm’s performance, we determined the OOB error rates for both models. In our sample, the OOB error rate improved from 0.32 in model 1 to 0.18 in model 2.

**Table 3:** Predictive performance measures for random forest models 1^a^ and 2^b^

Performance measures	Model 1	Model 2
Accuracy	0.69	0.82
*F*_1_-score^c^	0.28	0.55
Area under the curve	0.64 (receiver operating characteristic)/0.33 (precision-recall)	0.83 (receiver operating characteristic)/0.65 (precision-recall)
Sensitivity	0.25	0.45
Specificity	0.83	0.94
Positive predictive value	0.31	0.71
Negative predictive value	0.77	0.84
No information rate	0.76	0.76
Out-of-bag error rate	0.32	0.18

^a^Prediction of adherence based on sociodemographic and clinical variables.

^b^Prediction of adherence by adding the usage behavior within the first week.

^c^Harmonic mean between precision and recall.

### Predictor Importance

**Figure 5:**
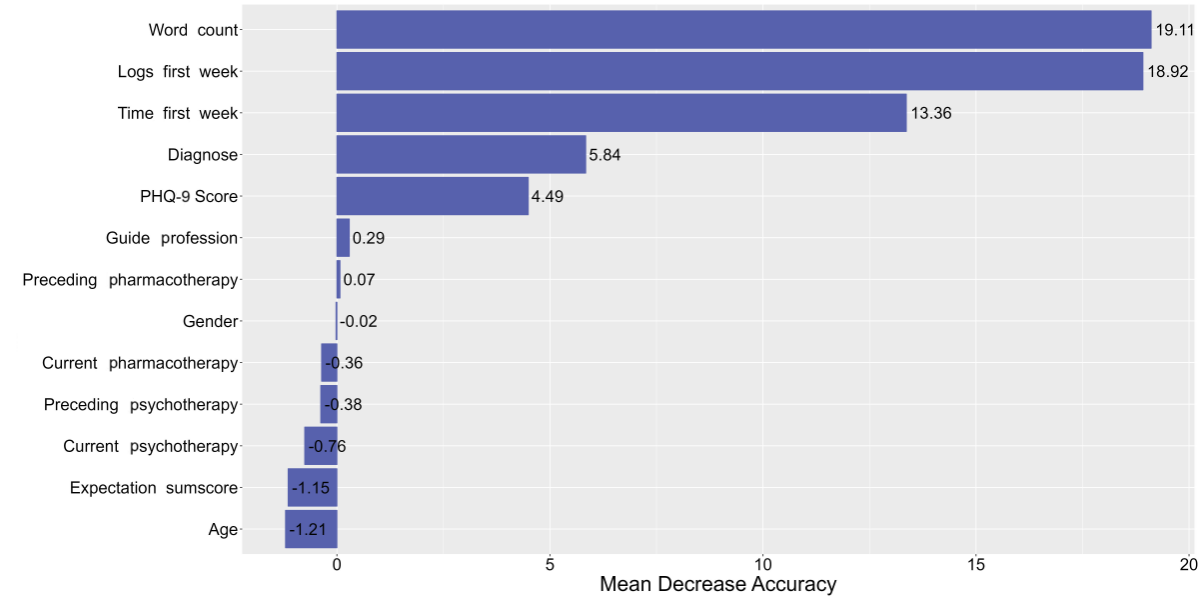
Variable importance ranking within model 2 (sociodemographic, clinical, and usage behavior predictors) sorted by predictor type for mean decrease accuracy. The x-axis shows the mean decrease accuracy which represents the loss of accuracy within the model, if the variable is excluded. The y-axis demonstrates the predictors sorted in the descending order. PHQ-9: 9-item Patient Health Questionnaire.

**Figure 6:**
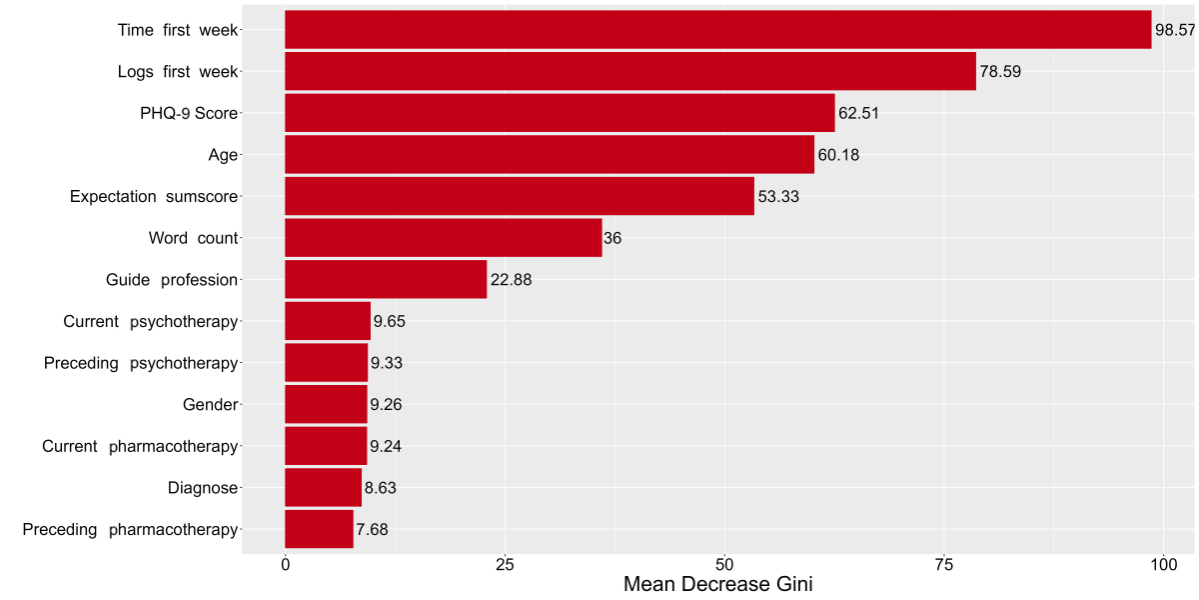
Variable importance ranking within model 2 (sociodemographic, clinical, and usage behavior predictors) sorted by predictor type for mean decrease Gini. The x-axis represents the mean decrease Gini which serves as a measure to determine to what extent each variable contributes to the homogeneity of the nodes in the resulting random forest. The y-axis demonstrates the predictors sorted in the descending order.

**Figure 7:**
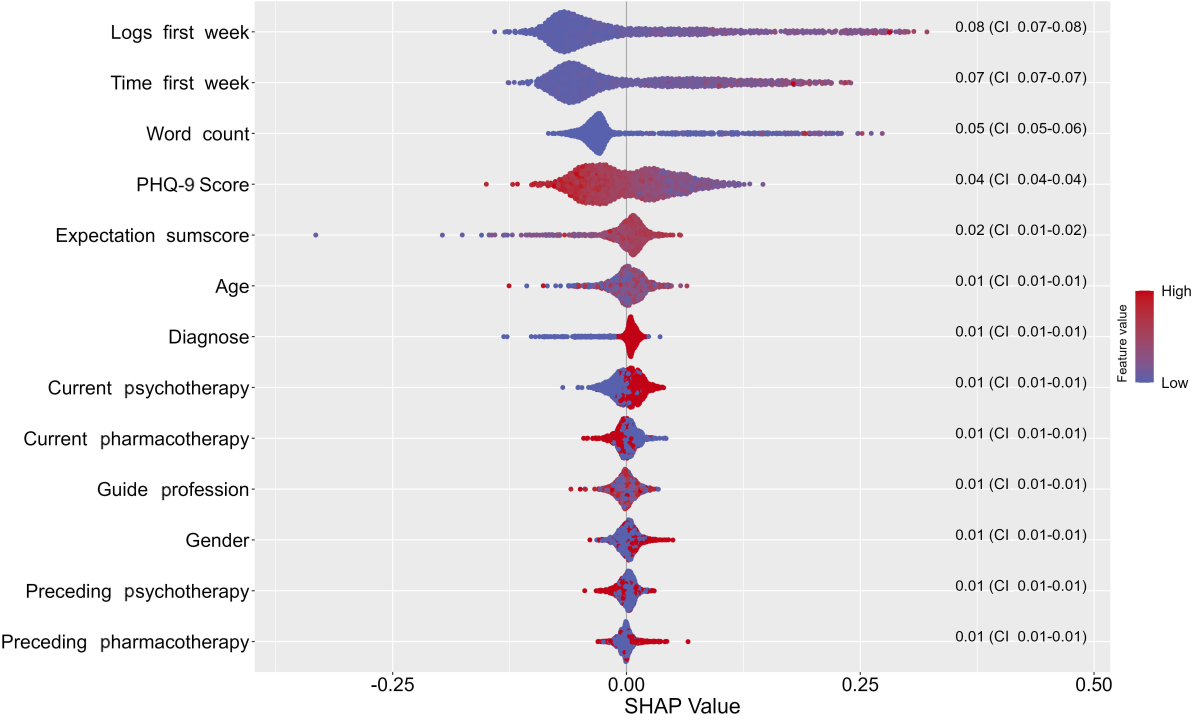
Bee swarm plot of Shapley Additive Explanation (SHAP) values of our predictor variables for adherence within model 2 (sociodemographic, clinical, and usage behavior predictors). A bee swarm plot is presented, with the x-axis representing the SHAP values and the y-axis displaying the predictors sorted in the descending order. Negative SHAP values indicate a negative influence on adherence, while positive values suggest a positive influence. For binary and categorical variables, the predictors are ranked in alphabetical order. Categorical variables are guide profession (low to high), including psychiatrist, general practitioner, psychotherapist, and others; gender (low to high), which includes female and male; binary variables (low to high), including false and true. PHQ-9: 9-item Patient Health Questionnaire.

As described above, predictor importance was assessed using mean decrease accuracy and mean decrease Gini, along with SHAP. Figures 5 and 6 illustrate the first 2 methods. The higher each variable appears in the rankings, the greater its importance in the prediction model. Notably, the 3 most important predictors based on mean decrease accuracy were all related to usage behavior. The word count from the first workshop’s worksheet during the initial week emerged as the most important predictor regarding mean decrease accuracy, followed by the logs from the first week and the time spent on the tool during that same period. The predictor importance according to SHAP values is displayed in Figure 7. This figure illustrates the order of importance, showing how each feature value of the predictors influences the prediction of the outcome measure (adherence), indicating whether a feature value has a positive or negative effect on adherence. In our case, we found that a high feature value for the number of logs within the first week and the number of seconds spent on the tool positively influenced adherence. Additionally, a higher expectation sum score and a lower PHQ-9 score were associated with increased adherence.

### Sensitivity Test and Subgroup Analyses

Out of the 1019 adherent participants, 124 (12.17%) met all prerequisites within the first week of their initial log-in to the iFD tool. Excluding these participants from the prediction yielded results similar to those in the main analysis (model 2). The accuracy was 0.81 (95% CI 0.79-0.83), with a specificity of 0.94. However, the *F*_1_-score was lower than in the complete sample, at 0.47, along with a sensitivity of 0.37 and a reduced positive predictive value. Because of the low internal consistency of the expectation sum score predictor, we conducted a second sensitivity test to rule out spurious results by excluding this predictor. This also yielded results similar to those in our main analysis. In addition to our sensitivity testing, we performed several subgroup analyses examining the impacts of gender, potential COVID-19 pandemic–related influences, various accompanying guide professions, and individual effects of clinical, sociodemographic, and usage behavior variables. The results and performance measures from these analyses can be found in Multimedia Appendix 1.

## Discussion

### Principal Findings

This study explored a random forest–based approach to predict adherence to the iFD tool, utilizing sociodemographic, clinical, and usage-related variables for a sample of 4187 adult routine care users. Our findings indicate that sociodemographic and clinical variables available at the beginning of the online intervention are insufficient to significantly predict user adherence to the iFD tool. Additionally, our predictor importance analyses revealed that all sociodemographic and clinical variables ranked lower than usage-related variables. The influence of sociodemographic factors as predictors of adherence has been inconsistent in previous studies. For instance, Beatty and Binnion [42] found no clear sociodemographic predictors for increased adherence, except for the user’s age. This finding is supported by Castro et al [43], who concluded that sociodemographic characteristics were not associated with adherence in their sample, and by Karyotaki et al [44], who reported that sociodemographic data had no predictive value in an individual patient data meta-analysis. Conversely, earlier research has indicated that patients’ sociodemographic characteristics are significant predictors of intervention usage, as demonstrated by Kazlauskas et al [45] in an intervention for adjustment disorder. In our sample, the nonadherent group had a lower mean age than the adherent group, reflecting similar findings in Karyotaki et al [16], which indicated that younger age increases the risk of nonadherence. However, age did not contribute meaningfully to the prediction of adherence. This suggests that while age may be associated with adherence, its predictive value is insufficient to warrant adjustments to intervention strategies. Similar to age, gender was indicative of adherence, but it played only a minor role as an actual predictor in our model. In our sample, there were more female participants in all 3 groups, which aligns with the findings of Karyotaki et al [16], who identified male gender as a risk factor for nonadherence, with the highest percentage of male participants found in the nonadherent group. These findings are consistent with previous research indicating that women tend to engage more in health-related behaviors than men [46]. It is important to note that this gender difference may be influenced by the format of the therapy (online vs in-person). Strauss et al [47] demonstrated that men were more likely to adhere to face-to-face therapy, suggesting that adherence varies by gender depending on how the intervention is delivered. The pretreatment severity score, which indicates the patient’s perceived depressive state, had one of the most significant impacts among nonusage-related variables in our model for predicting adherence, consistently ranking high in all predictor importance analyses. In our study, the severity measured by the PHQ-9 score was highest in the nonadherent group and lowest in the adherent group. This indicates that participants who perceived their depressive symptoms to be worse (ie, higher scores) at baseline were more likely to be nonadherent. This trend was also evident in our SHAP analysis, where higher feature values corresponded to negative SHAP values, indicating a negative influence on adherence. These findings contradict previous studies suggesting that worse symptoms at baseline promote continued use of the intervention. Fuhr et al [15] found that worse depressive symptoms (ie, higher scores) were predictive of higher adherence levels. Additionally, Wright et al [9] demonstrated that the largest mean effects in symptom improvement were observed in studies with high pretreatment severity scores. A possible explanation for this could be that patients with lower depressive symptoms are better equipped to navigate an online intervention such as the iFD tool independently. By contrast, more severely affected patients may struggle to engage with the online program due to the nature of their illness, particularly without external support from a therapist [48]. Another variable examined was outcome expectation, assessed through the entry questionnaire. In our study, the highest expectation sum score was found in the adherent group, which is consistent with previous findings indicating that increased outcome expectations may enhance adherence and persistence within online interventions [49]. However, it had less influence as a predictor of adherence in the importance ranking of mean decrease accuracy, ranking fifth in both the SHAP importance analysis and the mean decrease Gini analysis. The results regarding the profession of the guiding health care professional align with earlier findings [50], showing that guidance by a psychotherapist is associated with a higher likelihood of adherence. In our sample, 25.76% (438/1700) of participants met the adherence criteria when guided by a psychotherapist, compared with 21.43% (281/1311) when guided by a GP and 22.3% (116/520) when guided by a psychiatrist. However, these new findings provide context and indicate that the predictive importance of the guiding profession is relatively low compared with the usage behavior–related variables assessed in our analysis.

The time point for predicting adherence was shifted from before the intervention began to 1 week after its initiation. This change allowed us to incorporate usage behavior during the first week as an additional set of linguistic predictors alongside the previously established variables. The random forest algorithm subsequently identified this set of predictors as the most valuable components in the adherence prediction model, enabling us to significantly forecast adherence within the iFD tool. Our findings align with previous research indicating that linguistic predictors can effectively predict adherence, thereby enhancing the ability to determine adherence to an intervention [51]. In the meta-analysis conducted by Donkin et al [52], various linguistic predictors of adherence were examined. While their study found no relationship between log-ins and the outcomes of interventions targeting depression, our data indicate that log-ins are of great predictive value for adherence to the online intervention. Conversely, a prior study examining adherence to a web-based weight loss intervention found that early and extensive use of the intervention (specifically within the first 24 hours) was predictive of higher adherence, which supports our findings [53]. This suggests that predictions regarding a participant’s likely adherence based on our set of predictors can only be made after the intervention has commenced, rather than before, as previously suspected before our data evaluation. Our primary goal with the prediction was to identify nonadherent users to provide them with additional support. By incorporating linguistic variables, we improved specificity to 94% in our testing data. This means that the algorithm correctly identified 94.1% (894/950) of all nonadherent participants, missing only 56 out of 950 nonadherent individuals. By accurately identifying the majority of nonadherent participants after 1 week on the iFD tool they could be offered early, additional targeted support through various resources, such as reminder emails, SMS text messages, telephone calls, or face-to-face discussions with their accompanying physician or psychotherapist. This could enhance adherence to the tool, potentially reducing dropout rates and increasing the overall effectiveness of the intervention. A previous study suggests that the mode of delivery can influence the outcome, in our case the continuation of the intervention [9]. While impersonal forms of communication, such as SMS text messages or emails, yielded the lowest mean effects, telephone or face-to-face support demonstrated significantly greater impacts. The most effective form of support was in-person support from a clinician or another helping professional, particularly when the provider was a trained specialist, such as a psychiatrist [12]. Therefore, the optimal form of support for potential nonadherent participants in the iFD tool would be a face-to-face discussion with the prescribing health care provider after the first week of the intervention, allowing clinicians to allocate resources more efficiently. Rather than waiting for adherence problems to arise, resources can be directed toward patients who need them the most, potentially optimizing the use of time and effort in clinical practice. In our predicted test data set, 167 out of 1255 participants (13.31%) were classified as false positives, that is, these participants were classified as nonadherent although they were adherent according to our definition. These participants would still receive the additional support without the need for it. As face-to-face support is time and cost-intensive, further investigations need to be conducted to find the most appropriate support while considering optimal resource utilization.

Looking at the adherence within the iFD tool in general, the adherence rate (1019/4187, 24.34%) in our sample was lower than that reported in other studies. Richards and Richardson [54] reported adherence rates of 62% for interventions with administrative support and 72% for those in which the patient received support from a therapist. Examining adherence in routine care settings, Etzelmueller et al [12] found similar results, with adherence rates of 61.3% for completing the iCBT interventions as planned. A possible explanation for the discrepancy between our results and those mentioned above is that adherence is defined differently in each study, depending on its application and feasibility. In our study, adherence was defined as a combination of personal progress and active engagement, measured through the completion of workshops within the tool and the number of log-ins. By contrast, other studies defined adherence solely based on the completion of workshops [12,55]. Another explanation for the low adherence and completion rates observed in our online intervention may be that the iFD tool is currently available only as a browser version. A recent study found that low completion rates may be attributed to a lack of engagement with the computer program itself [9]. Factors contributing to this lack of engagement could include issues accessing the online tool (such as limited internet connectivity or mobility constraints) or a general lack of experience with online resources (related to age or education level) [9]. One potential solution to these issues is to improve the usability of the intervention, for example, by developing a mobile app version. Apps are easily accessible, particularly for patients with limited access to conventional therapy, such as those living in rural areas. They can also be used offline, minimizing potential difficulties in accessing the tool [56]. Furthermore, research has demonstrated that smartphone apps for depression can be effective in alleviating depressive symptoms, achieving modest overall effects [57]. Another potential solution is to incorporate more engaging features, such as gamification—the process of adding gaming elements such as levels and reward systems to a nongame context to enhance participation—into the online intervention. Its primary aim is to increase adherence and, consequently, motivation within the intervention [58]. To our knowledge, there is limited research on the impact of gamification on adherence in mental health apps specifically designed for treating depression. Wahle et al [59] found that incorporating feedback mechanisms on progress during the intervention as a game element significantly reduced depressive symptoms. In general, when examining the effects of mental well-being apps, such as the eQuoo app (a mobile mental health game), gamification has been associated with a higher adherence rate compared with nongamified well-being apps (control group), achieving an adherence rate of 90% [56].

### Strength and Limitations

The strength of this study lies in its large sample size and the data collected from routine care. We believe that our sample is unaffected by potential study effects and represents a diverse range of individuals of different ages, genders, and stages of depression. Additionally, because the iFD tool is based on CBT, our findings may be transferable to comparable available interventions targeting depression, such as moodgym or deprexis.

Our study has several limitations. First, our definition of adherence does not follow the strictest standard, which would involve the completion of all workshops [60] for our personal progress criterion. This cutoff for defining adherence was not feasible in our study due to the low adherence rate, which would have resulted in a greater imbalance between the adherent and nonadherent groups. Additionally, the impact of the intervention on depressive symptoms can begin before all provided workshops are completed. Consequently, we adapted our individual criteria to fit the nature of our intervention, which differed in duration, structure, and other aspects from other interventions. This approach allowed us to create a tailored adherence metric that accurately reflected participants’ interactions with our intervention.

Second, in our study, the word count was determined solely for the first worksheet and did not take into account the following worksheets in the subsequent workshops. We chose this measure to establish a predictor that is easy to implement and can be used in everyday clinical practice to identify risk groups as early as possible within the intervention. Another associated limitation is that 2691 out of 4187 participants either did not use the worksheets or opted to print them out and fill them in manually, resulting in a lack of information regarding the word count for these individuals. Consequently, the word count for these participants was recorded as N=0 (a conservative/negative estimate) when training our random forest model.

Third, this study examined only a limited number of potential predictors for adherence. All data, except for the usage-related variables, were collected through a brief entry questionnaire at the beginning of the iFD tool, which was designed to gather only essential information about the user. Previous studies have identified additional sociodemographic predictors, such as income and marital status, as well as clinical predictors, such as comorbidity, that may influence adherence to the intervention. Future research could explore whether including these variables in the entry questionnaire would enhance the prediction of adherence within the iFD tool.

Fourth, the analysis presented in this paper is based solely on a single machine learning algorithm, specifically random forest. This choice was made as a proof of concept for a machine learning–based adherence prediction algorithm tailored to our specific research question. Future research could incorporate comparative analyses between different machine learning methods in subsequent studies.

Fifth, due to the low internal consistency of the predictor expectation sum score (Cronbach α=0.61), there may be an underestimation of the effect of expectations regarding the intervention on adherence.

Lastly, our sample is limited to Germany and the German language, which may not adequately capture potential cultural differences related to adherence and behavior toward the intervention. Therefore, future studies could focus on comparing these results with samples of the iFD tool from different countries and languages once implementation has progressed in those regions.

### Conclusions

This study focused on predicting patient adherence to the iFD tool. Adherence can be predicted using usage behavior within the first week of the intervention. With this information, it becomes possible to identify, at an early stage, users who are likely to process the online intervention incompletely or not at all due to their characteristics, thereby reducing their chances of benefiting from the iFD tool. A next step could be to develop an automated monitoring program based on the built random forest model to analyze the usage behavior during the first week of the intervention for each participant. This program could initiate targeted additional support for nonadherent participants. In this way, our machine learning approach can improve care for patients with depression through online interventions by facilitating the early recognition of nonadherence and possibly enabling proactive measures to improve it.

## Appendix

Multimedia Appendix 1 Random forest–based prediction of adherence.

## Abbreviations

AUC: area under the curve
CBT: cognitive behavioral therapy
DSM-IV: Diagnostic and Statistical Manual of Mental Disorders, fourth edition
EAAD: European Alliance Against Depression
GP: general practitioner
iCBT: internet-based cognitive behavioral therapy
iFD: iFightDepression
OOB: out of bag
PHQ-9: 9-item Patient Health Questionnaire
PR: precision-recall
ROC: receiver operating characteristic
SHAP: Shapley Additive Explanations
TRIPOD: Transparent Reporting of a Multivariable Prediction Model for Individual Prognosis or Diagnosis

## Data Availability

The data set and code used for conducting this study are available from the corresponding author upon reasonable request.
