# Supplementary material for: Use of Random Forest to Predict Adherence in an Online Intervention for Depression Using Baseline and Early Usage Data: Model Development and Validation on Retrospective Routine Care Log Data
Source: JMIR Form Res. 2024 Nov 15;8:e53768. doi: 10.2196/53768 (PMC11607565; doi:10.2196/53768)
Supplement: Multimedia Appendix 1 [file formative_v8i1e53768_app1.pdf]

https://doi.org/10.2196/preprints.53768

**Mulitmedia Appendix:**

Subgroup analyses:

1. Comparison between random forest models predicting adherence to the iFightDepression tool (iFD tool) separate for female and male participants to identify possible differences in performance between gender found no significant differences within our analysis ($t(1100.74) = 1.62$, $p = .106$).

| **Female (2537 participants / Overall adherence rate: 24.60%)** | | | | | | | | |
|---|---|---|---|---|---|---|---|---|
| ACC | F1 | AUC | Sensitivity | Specificity | PPV | NPV | NIR | OOB |
| 0.83 | 0.63 | 0.83 (ROC)/ 0.69 (PR) | 0.58 | 0.92 | 0.69 | 0.87 | 0.75 | 0.19 |
| **Male (1640 participants / Overall adherence rate: 24.00%)** | | | | | | | | |
| ACC | F1 | AUC | Sensitivity | Specificity | PPV | NPV | NIR | OOB |
| 0.81 | 0.53 | 0.83 (ROC)/ 0.65 (PR) | 0.45 | 0.92 | 0.64 | 0.84 | 0.76 | 0.20 |

Table S1. Performance measures for the subgroups female and male only
ACC: Accuracy
F1: Harmonic mean between precision and recall (F1-score)
AUC: Area under the curve
PPV: Positive predictive value
NPV: Negative predictive value
NIR: No Information Rate
OOB: Out-of-bag error rate

2. Comparison between pre-COVID and during-COVID participants of the iFD tool to identify possible differences in the performance of the random forest models due to possible COVID-19 pandemic-related influences found no significant differences within our analysis ($t(1031.11) = 0.75$, $p = .455$).

| **Pre-COVID (1655 participants)** | | | | | | | | |
|---|---|---|---|---|---|---|---|---|
| ACC | F1 | AUC | Sensitivity | Specificity | PPV | NPV | NIR | OOB |
| 0.79 | 0.53 | 0.79 (ROC)/ 0.61 (PR) | 0.46 | 0.91 | 0.63 | 0.83 | 0.74 | 0.21 |
| **During-COVID (2532 participants)** | | | | | | | | |
| ACC | F1 | AUC | Sensitivity | Specificity | PPV | NPV | NIR | OOB |
| 0.84 | 0.60 | 0.84 (ROC)/ 0.67 (PR) | 0.52 | 0.93 | 0.70 | 0.86 | 0.77 | 0.18 |

Table S2. Performance measures for the subgroups pre-COVID and during-COVID

https://doi.org/10.2196/preprints.53768

3. Performance measures of the main random forest analysis predicting adherence to the iFD tool, considering only one variable group at a time as a set of predictors. Compared to the full model, all three subgroups displayed significant differences in performance, with all of them scoring lower compared to the full model.
    - Usage behaviour only: $t(2476.26) = 3.57$, $p < .001$
    - Clinical variables only: $t(2388.13) = 31.65$, $p < .001$
    - Sociodemographic variables only: $t(2305.73) = 18.32$, $p < .001$

| **Usage behaviour only** | | | | | | | | |
|---|---|---|---|---|---|---|---|---|
| ACC | F1 | AUC | Sensitivity | Specificity | PPV | NPV | NIR | OOB |
| 0.77 | 0.51 | 0.78 (ROC)/ 0.63 (PR) | 0.48 | 0.87 | 0.54 | 0.84 | 0.76 | 0.22 |
| **Clinical variables only** | | | | | | | | |
| ACC | F1 | AUC | Sensitivity | Specificity | PPV | NPV | NIR | OOB |
| 0.45 | 0.41 | 0.59 (ROC)/ 0.33 (PR) | 0.79 | 0.34 | 0.28 | 0.84 | 0.76 | 0.63 |
| **Sociodemographic variables only** | | | | | | | | |
| ACC | F1 | AUC | Sensitivity | Specificity | PPV | NPV | NIR | OOB |
| 0.52 | 0.33 | 0.56 (ROC)/ 0.29 (PR) | 0.50 | 0.52 | 0.25 | 0.76 | 0.76 | 0.49 |

Table S3. Performance measures for the subgroups usage behaviour, clinical variables and sociodemographic variables only

https://doi.org/10.2196/preprints.53768

4. Comparison between participants, who received guidance within the iFD tool through a General Practitioner, Psychiatrist or Psychotherapist found no significant differences between these three groups within our analysis ($F(2.11) = 2.11$, $p = .122$).

| **General Practitioner (GP)** | | | | | | | | |
|---|---|---|---|---|---|---|---|---|
| ACC | F1 | AUC | Sensitivity | Specificity | PPV | NPV | NIR | OOB |
| 0.86 | 0.58 | 0.86 (ROC)/ 0.65 (PR) | 0.45 | 0.97 | 0.81 | 0.87 | 0.79 | 0.17 |
| **Psychiatrist** | | | | | | | | |
| ACC | F1 | AUC | Sensitivity | Specificity | PPV | NPV | NIR | OOB |
| 0.82 | 0.50 | 0.82 (ROC)/ 0.64 (PR) | 0.41 | 0.93 | 0.64 | 0.85 | 0.78 | 0.18 |
| **Psychotherapist** | | | | | | | | |
| ACC | F1 | AUC | Sensitivity | Specificity | PPV | NPV | NIR | OOB |
| 0.79 | 0.51 | 0.78 (ROC)/ 0.57 (PR) | 0.43 | 0.91 | 0.62 | 0.82 | 0.74 | 0.21 |

Table S4. Performance measures for the subgroups guided by a General Practitioner, by a Psychiatrist or by a Psychotherapist

5. When examining the influence of the “guide profession” on the meaningfulness of the set of clinical variables, a significant difference was found depending on whether the “guide profession” was included in the clinical variables or not ($t(2174.38) = -13.13$, $p < .001$).

| **Clinical variables (without Guide profession)** | | | | | | | | |
|---|---|---|---|---|---|---|---|---|
| ACC | F1 | AUC | Sensitivity | Specificity | PPV | NPV | NIR | OOB |
| 0.31 | 0.40 | 0.56 (ROC)/ 0.30 (PR) | 0.95 | 0.11 | 0.25 | 0.87 | 0.76 | 0.71 |
| **Clinical variables (with Guide profession)** | | | | | | | | |
| ACC | F1 | AUC | Sensitivity | Specificity | PPV | NPV | NIR | OOB |
| 0.45 | 0.41 | 0.59 (ROC)/ 0.33 (PR) | 0.79 | 0.34 | 0.28 | 0.84 | 0.76 | 0.63 |

Table S5. Performance measures of the clinical variables with and without the “guide profession”
